# Co-Creating an Intervention to Prevent Injuries in Police Force Recruits: A Concept Mapping Study of Police Force Recruits, Police Force Staff, Health Professionals, and Research Experts

**DOI:** 10.1186/s40798-026-01042-9

**Published:** 2026-06-28

**Authors:** Myles Murphy, Andrea B. Mosler, Garth Allen, Alex Donaldson, Clare L. Ardern, Carolyn A. Emery, Martin Hägglund, Jonathan M. Hodgson, Joanne L. Kemp, Debra Langridge, Tiffany McAlinden, Thomas Mirco, Sophia Nimphius, Simone Radavelli-Bagatini, Kiera Staley, Vanessa Sutton, Evert Verhagen, Andrea M. Bruder

**Affiliations:** 1 Nutrition and Health Innovation Research Institute, Edith Cowan University, Joondalup, WA Australia; 2 Institute for Health Research, The University of Notre Dame Australia, Fremantle, WA Australia; 3 La Trobe Sport and Exercise Medicine Research Centre, La Trobe University, Bundoora, VIC Australia; 4 Australian International Olympic Committee Research Centre, Melbourne, VIC Australia; 5 Physical Performance Unit, Western Australia Police Force, Joondalup, WA Australia; 6 Centre for Sport and Social Impact, La Trobe University, Melbourne, VIC Australia; 7 Department of Physical Therapy, The University of British Columbia, Surrey, Canada; 8 Centre for Aging SMART, The University of British Columbia, Vancouver, Canada; 9 Sport Injury Prevention Research Centre, Faculty of Kinesiology, University of Calgary, Calgary, AB Canada; 10 Department of Health, Medicine and Caring Sciences, Unit of Physiotherapy, Linköping University, Linköping, Sweden; 11 Consumer and Community Involvement Program, University of Western Australia, Nedlands, WA Australia; 12 Patient and Public Involvement Representative, Western Australia Police Force, Perth, WA Australia; 13 School of Medical and Health Sciences, Edith Cowan University, Joondalup, WA Australia; 14 Amsterdam Collaboration on Health and Safety in Sports, Department of Public and Occupational Health, Amsterdam Movement Sciences, Amsterdam UMC, Amsterdam, Netherlands; 15 Department of Physiotherapy, Podiatry, Prosthetics and Orthotics, La Trobe University, Bundoora, VIC Australia

**Keywords:** Law enforcement, Tactical athletes, Occupational health, injury prevention, participatory, Consumer involvement

## Abstract

**Objective:**

Police force recruits have a high musculoskeletal injury burden, which results in a substantial economic burden and can lead to attrition. The objective of this study was to identify and prioritise the strategies perceived as important and feasible to reduce the prevalence, incidence, and burden of injury in police force recruits.

**Design:**

Mixed-methods concept mapping study.

**Methods:**

Forty-eight participants were recruited from four broad groups: police force recruits/officers; police force staff; health professionals; and research experts. Participants brainstormed statements in response to a prompt (“To prevent injury and/or reduce the impact of injury on law enforcement recruit training, I think it’s important to….”) before sorting and rating the statements/strategies for importance and feasibility. Descriptive statistics, multi-dimensional scaling, hierarchical cluster analysis, pattern matching and Welch’s t-tests were applied.

**Results:**

Ninety-six unique prevention strategies were identified (42 were above the grand mean for both importance and feasibility). Eight clusters appropriately represented all statements. From highest to lowest mean cluster importance these were: i) clearly communicate physical training program expectations and requirements; ii) prepare for, monitor and manage physical training load; iii) provide best practice injury identification, prevention and management; iv) educate recruits, staff and other stakeholders involved in academy training delivery; v) provide a supportive training environment that promotes health, wellbeing and injury reporting; vi) have experienced staff deliver training and use appropriate equipment; vii) deliver a comprehensive and holistic physical training program; and viii) have appropriate physical entry standards and requirements.

**Conclusion:**

We identified 42 strategies above the grand means for both importance and feasibility to reduce the burden of injury in police force recruits. These strategies can be implemented by recruits, staff delivering the training program, and/ or staff managing or governing the training program. Future research should refine how these strategies can be implemented in practice and policy.

## Introduction

Due to challenges in recruitment, police force academies around the world have lowered their physical entry standards as a strategy to increase recruit numbers [1, 2]. Therefore, it is essential that recruits who enter training remain during the training program and graduate. However, one in twenty law enforcement recruits in the United States of America drop out of their recruit training program due to injury [3]. Drop-out rates in Australia are unknown. However, one in five recruits within an Australian state police force suffer a musculoskeletal injury during the recruit training program, resulting in time-loss from work [4, 5]. The reported time-loss injury incidence rate of 2.0 injuries per 1000 training days [4] is comparable to other law enforcement and military recruit populations worldwide [3, 6]. The primary regions of injury in police force recruits are the lower limb (e.g., ankle, knee and hip) and the shoulder, similar to those observed in military recruits [4, 7]. Police force recruit injuries predominantly occur in joint, ligament, muscle and tendon tissue types [4, 5]. In contrast, bone stress injury is far more common and burdensome in military recruit populations [7].

Several factors have been proposed as potential contributors to injury in police force recruits [8]. However, most of these proposed risk factors are supported by very low certainty evidence [8]. Older age (> 30 years) and female sex are the most consistently reported demographic variables associated with a higher overall injury incidence rate [8]. Lower baseline cardiorespiratory fitness, as measured by running-related performance outcomes (e.g., beep test, 1.5-mile run test) and lower baseline physical endurance capacity (e.g., pull-up repetitions to failure, push-up repetitions to failure, sit-up repetitions to failure) are also associated with higher injury rates [8]. These findings suggest that better cardiorespiratory fitness and physical endurance may have a protective effect. No psychological variables have been evaluated in relation to injury incidence among police force recruits [8].

It is widely accepted that evidence-informed strategies within the hierarchy of control [9] are effective in reducing occupational injury. However, in a police force recruit training environment both elimination and substitution of physical training is not always possible. There is almost no research on the efficacy of prevention interventions in police force recruits to guide everyday practice. There is some evidence for engineering controls, with one study in female Israeli border police recruits, which explored two different types of vests and found that recruits wearing lighter vests had a lower incidence of bone stress injury [10]. Only one other study has explored injury prevention in police force recruits. This study implemented the administrative control of standard versus ability-based training in police force recruits, with no significant differences in injury incidence between groups [11]. Despite substantial injury burdens, and more than 30 studies examining factors associated with injury [8], scientific evidence for how injuries in police force recruits can be prevented is scarce. In the absence of empirical evidence, our study compiled content and context expert perspectives on what may be effective to reduce the burden of injury in police force recruits to assist in the development of future prevention interventions.

To move beyond the epidemiology and aetiology of injury in police force recruit populations, our concept mapping approach identified and prioritised the strategies perceived by those with lived experience of recruit training and/ or delivery, healthcare provision or research expertise as most important and feasible for implementation as interventions to reduce injury burden in police force recruits.

The objective of our study was to identify and prioritise the key strategies perceived as important and feasible to reduce the prevalence, incidence, and burden of injuries among police force recruits. We engaged police force recruits or officers, police force staff, health professionals, and research experts to achieve our objective.

## Methods

We used online Concept Mapping within a participatory action research approach [12], adapted from our previous approaches to developing injury prevention interventions [13–15]. Concept mapping is a valid and reliable mixed-methods approach to generate, organise and conceptualise unexplored concepts with diverse participant perspectives [16]. In our study, the concept mapping approach was supported by consumer and industry partners [17], and informed by previously identified and synthesised evidence [3–5, 8, 18]. The concept mapping exercise asked participants to generate, sort and rate ideas in response to a study focus prompt, followed by analysis, interpretation and use/application of the results [19].

### Consumer and Industry Contribution

Our study was undertaken in accordance with our overall governance structure. Specifically, we have an overall steering committee, which is supported via a consumer advisory group, industry advisory group, and research advisory group [17]. The advisory groups make recommendations (typically related to project aims, methods, analysis, interpretation or results, dissemination and translation), which are then actioned, where possible, by the steering committee.

### Prospective Registration

Our protocol outlining the concept mapping approach and our consumer and industry partner involvement was prospectively registered [17, 20], and this study has been reported according to the ConMapT guidelines [21]. All non-identifiable data for this research project are available online (10.6084/m9.figshare.30486179).

### Ethical Considerations

The Edith Cowan University Human Research Ethics Committee approved this research (approval number: 2024-05919-MURPHY), and the La Trobe University Human Research Ethics Committee provided reciprocal approval. Participants provided informed electronic consent via an online Qualtrics survey (Qualtrics, Provo, UT).

### Participants

We aimed to recruit approximately 50 participants across four groups: police force recruits and officers; police force staff; health professionals; and research experts. The diverse groups helped to ensure that all major perspectives on the topic were represented [22]. The police force recruits or officers included adults over 18 years of age, from diverse genders, who had worked in the police force in the previous 12 months and/or had sustained an injury during tactical training that resulted in an inability or impaired ability to work. The police force staff included people who administered and delivered police force recruit training (e.g., physical trainers, police instructors, health and wellness staff, or executive staff). Health professionals included people with experience in preventing and managing musculoskeletal injuries (e.g., dietitians, exercise physiologists, general practitioners, orthopaedic surgeons, physiotherapists, psychologists, sports medicine physicians, strength and conditioning coaches). Research experts included researchers with publications in the field of musculoskeletal injury prevention in physically active populations (e.g., tactical or sporting populations). For healthcare professionals and research experts, we applied the closeness continuum for consideration for selection, which is becoming more common in consensus research [23], and meant that in some instances researchers may not have been invited (i.e. we limited how many researchers per collaboration/ institution could be included). This also meant research experts were not eligible for inclusion if they were on the research team.

Although there is no limit to the number of people who can participate in a concept mapping study [19], our previous research in this field suggests that between 40 to 50 participants in the brainstorming balances idea saturation with receiving an excess of redundant responses [24, 25]. Between 20 and 30 participants are recommended for the sorting activity to maximise the consistency of fit in the concept mapping representation by minimising the variability in the stress value found with smaller groups of sorters [16].

### Recruitment

Participants were recruited in March 2025 via purposive recruitment strategies, consistent with our desired sample size. Potential participants were invited via existing industry networks and professional organisations. Participants received a $50 gift card as an honorarium for their time and recognition of their expertise.

### Setting

Participants completed an online Qualtrics survey, which included capture of several demographic variables enabling us to describe the cohort. After completing the survey, participants received an email link to the concept mapping platform. All concept mapping data were collected and analysed using the Concept Systems Inc Groupwisdom™ software (Ithaca, NY).

### Demographic Data

The following data were collected using Qualtrics and are described in depth in Appendix A: age; sex; gender; body mass index (BMI); ethnicity; country of primary residence; languages other than English; participation in moderate to vigorous physical activity; education level; employment status; total household income; Nordic musculoskeletal screening [26, 27]; role; time in role; specific occupation; history as a tactical athlete (for police recruits and officers only); previous prevention project design (for research experts only).

### Concept Mapping Data

We employed concept mapping to identify the strategies perceived to be necessary to reduce the burden of injury among police force recruits. We followed key steps described by Kane and Trochim [19], which are detailed in Appendix B and summarised below. Participants were asked to respond to the study focus prompt: “To prevent injury and/or reduce the impact of injury on law enforcement recruit training, I think it’s important to….” Participants could brainstorm as many ideas as they wanted to and had access to the software for 10 days, with regular reminders. Participants could also see ideas that had already been brainstormed. After the brainstorming activity, the project steering committee (MM, KS, ABM, AD, AMB) synthesised the statements via an iterative process. Synthesis included splitting compound statements, deleting statements unrelated to the study prompt, removing duplicate or unclear statements and checking and editing statements to enhance clarity or grammar [24].

### Statement Sorting and Rating

Over 14 days, participants were asked to group the statements from the condensed list (those synthesised per the above procedure), which were presented in a random order, into 5–15 piles of related ideas and to name each pile based on its theme/contents. Participants were instructed to avoid grouping statements based on a value (e.g., a group of what they thought were most important), otherwise their response was classified as invalid. Participants were also instructed to avoid creating groups of unrelated statements (e.g., a group containing items they were unsure about), otherwise their response was classified as invalid. Participants were asked to rate each statement for importance and feasibility for inclusion in a recruit injury prevention program, using a five-point scale (i.e. 1 = least important/ feasible, 5 = most important/ feasible). Participants were instructed to rate each statement relative to other statements on the list and to use all item response categories (e.g., avoid assigning all items a rating of 1 or 5 only).

### Analysis and Interpretation

We used the Concept Systems Inc. Groupwisdom™ software (Ithaca, NY) platform to produce point maps (using multidimensional scaling, where each sorted statement represented a separate point) and cluster maps (using hierarchical cluster analysis). All analyses were conducted by the Concept Systems Inc. Groupwisdom™ software (Ithaca, NY) platform.

The research team iteratively reviewed the statement groups in the cluster maps in collaboration with the consumer and industry advisory groups. This also included amending the cluster boundary when an item appeared to have a better fit in an adjacent cluster. Boundary amendment was performed after selecting the most appropriate cluster map, and assessing the appropriateness of each individual statement within its assigned cluster. Where supported by the raw sorting data and bridging values (indicating how often a statement was sorted with nearby statements on the map), cluster boundaries were modified to reflect better conceptual fit. Therefore, the final cluster map was selected based on conceptual coherence (i.e. statement grouping makes sense and clusters are distinct), statistical indicators (i.e. examining bridging values to assess if statements are conceptually tight and indicate a strong cluster. Whereby lower values reflect tighter thematic grouping), and practical relevance (i.e. meaningful to end-users and relevant to intervention development) [17]. The final map solution was agreed upon during an online meeting with the consumer advisory group, the industry partner advisory group, and the research team. A stress index (between 0 and 1) was calculated to assess the goodness of fit between the point map and the original sorting data (lower values indicate better fit) [16].

Mean importance and feasibility ratings were calculated for each statement and plotted on a bivariate Go-Zone graph [19], a scatterplot divided into quadrants using the grand mean of each rating scale. This visualisation identified priority ideas (e.g. those rated above the grand mean on both scales, or high in importance but lower in feasibility). A Pearson correlation coefficient was used to measure the linear association between the two scales [17]. Pattern matching was used to visually compare mean cluster rating across participant subgroups (i.e. Police Force vs. Clinician/Researcher) [25]. When steep pattern match slopes suggested notable differences, Welch’s t-tests were applied to compare subgroup means within clusters. This test accounts for unequal variances and sample sizes, and cluster means were treated as interval-level data derived from item averages.

## Results

### Participants

Forty-eight people participated in this study [police force recruits or officers (*n* = 6), police force staff (*n* = 19), health professionals (*n* = 13), and researchers (*n* = 10)], with the participation breakdown by phase presented in Appendix B. A maximum of two health professionals or researchers shared a primary affiliation. Police force recruits or officers were either injured (*n* = 3), or uninjured (*n* = 3). Police force staff represented a variety of roles [Academy Managers (*n* = 5), Physical Training Instructors (*n* = 3), Police Force Health and Safety Officers (*n* = 3), Recruit Support Officers (*n* = 3), Use of Force and Weapons Instructors (*n* = 2), Performance Data Analyst (*n* = 1), Physiotherapist (*n* = 1), and Psychologist (*n* = 1)]. Various health professions were represented [general practice (*n* = 2), nutrition and dietetics (*n* = 2), physiotherapy (*n* = 2), podiatry (*n* = 2), sport and exercise medicine (*n* = 2), strength and conditioning (*n* = 1), biomechanics (*n* = 1), exercise physiology (*n* = 1)]. Researchers represented a variety of areas relevant to the police force [general injury prevention (*n* = 2), bone injury (*n* = 1), concussion (*n* = 1), knee Injury (*n* = 1), low back injury (*n* = 1), muscle/tendon injury (*n* = 1), orthopaedics (*n* = 1), nutrition and dietetics (*n* = 1), and psychology (*n* = 1)]. Of the health professionals and research experts: ten were from national level sport, seven were from private business, five were from universities, and one was from the Australian Defence Force. Complete participant demographics are presented in Table 1 and Appendix C.

**Table 1:** Participant characteristics

Domain	Variable	Police force recruit or officer (*n* = 6)	Police force staff (*n* = 19)	Health professional (*n* = 13)	Research expert (*n* = 10)
Sex	Female	3	10	8	7
	Male	3	9	5	3
Gender	Woman or female	3	10	8	7
	Man or male	3	9	5	3
Country	Australia	6	19	12	8
	Qatar	0	0	0	1
	Switzerland	0	0	1	0
	United Kingdom of Great Britain and Northern Ireland	0	0	0	1
Multi-lingual	Yes	0	3	4	4
	No	6	16	9	6
English first language if multi-lingual	Yes	0	1	3	1
	No	0	2	1	3
Self-reported regular moderate to vigorous physical activity	Yes	5	15	12	8
	No	1	4	1	2
Education	Less than high school degree	1	1	0	0
	High school graduate	3	2	0	0
	Bachelor's degree	2	8	3	0
	Master's degree	0	6	3	0
	Doctoral degree	0	2	4	9
	Professional degree (e.g., Medical Doctor)	0	0	3	1
Work status	Working full-time	6	17	10	10
	Working part-time	0	2	2	0
	Student	0	0	1	0
Income	Between AUD $50,000–$79,999	1	0	1	0
	Between AUD $80,000–$99,999	0	3	1	0
	Between AUD $100,000–$149,999	2	3	3	0
	Between AUD $150,000–$199,999	1	4	1	2
	More than AUD $200,000	2	9	7	8
Tactical Background	Yes	0	2	N/A	N/A
	No	6	17	N/A	N/A

Data are expressed as numbers for each variable

### Concept Mapping

Participants contributed 180 statements in the brainstorming activity. The steering committee then synthesised and refined the statements. Ninety-six unique statements were made available to participants for the sorting and rating activities (Appendix D).

### Statement Sorting and Cluster Solution

**Fig. 1:**
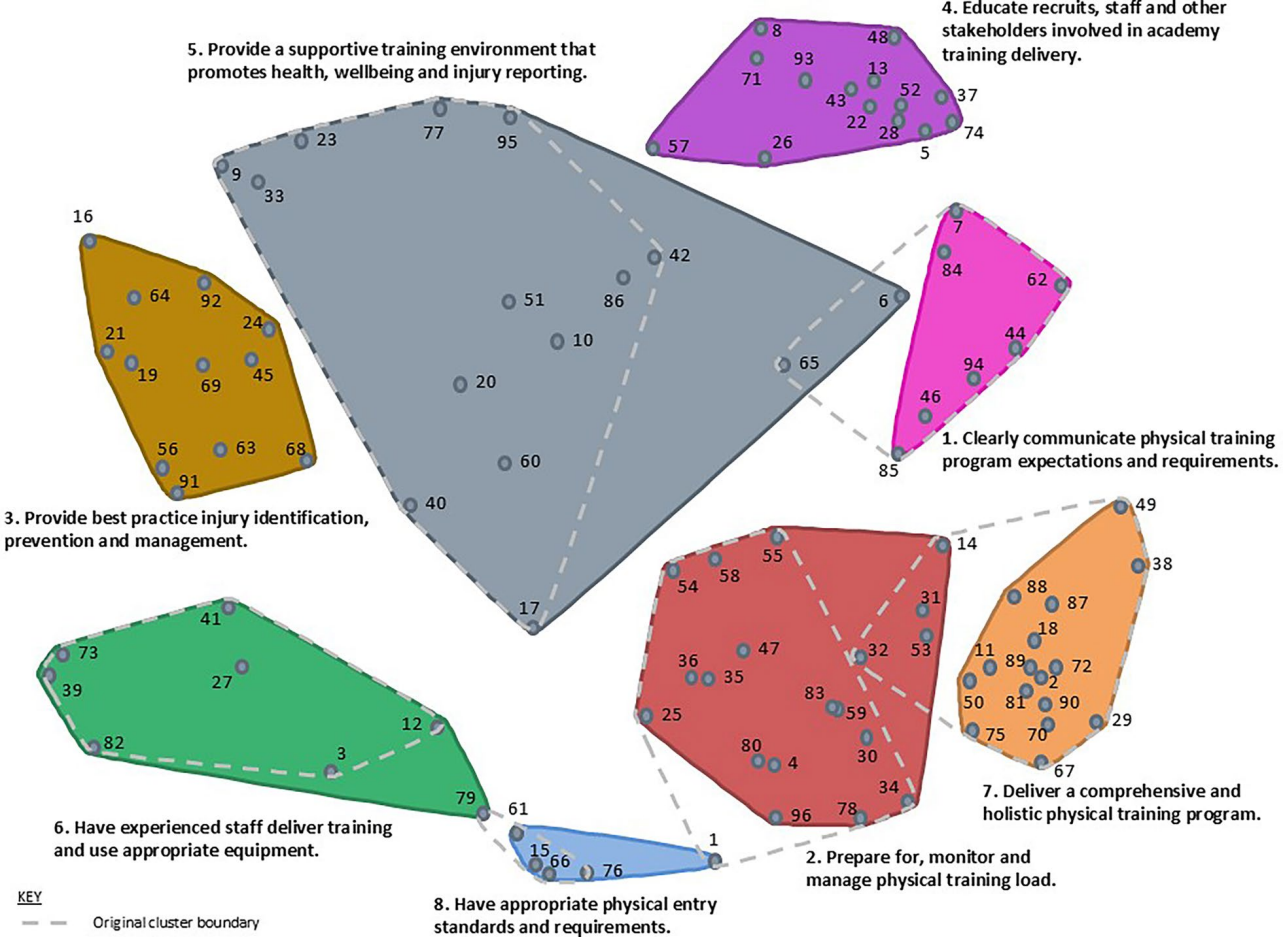
Optimal cluster mapping solution for how to prevent injury and/or reduce the impact of injury on police force recruit training. Legend: Hierarchical cluster analysis was used to produce point maps (using multidimensional scaling). Each sorted statement (*n* = 96) is represented by a separate point/ dot, and each cluster [1–8] is represented by a coloured shape. The dashed line represents the original cluster boundary before review and amendment by the steering committee, industry advisory group, and consumer advisory group

Forty-two participants sorted the statements (with valid responses from 36 included in the analysis: mean = 8.8 groups; mode = 7; range = 5–18). A range of cluster solutions (15 to 5) were generated and presented by a single study author (KS) to the project steering committee, who identified that an eight-cluster solution provided the most useful representation of statements (Stress Index = 0.2854, reflecting that participants’ accurately sorting data and results are unlikely to be random or without structure). Two study authors (MCM, AMB) then presented various cluster solutions (from a 10 cluster solution and down) to the consumer and industry advisory groups, who also selected an eight-cluster solution (Fig. 1) as the best representation of the participants’ sorting data. The steering committee provisionally named all eight clusters based on the content of the clusters and the names used by participants when creating groups of statements in the sorting activity. The cluster names were then amended after consulting with the consumer advisory group and the industry advisory groups (Appendix E).

A full list of the strategies within each cluster, including the eight statements reassigned to an adjacent cluster, is provided in Appendix D. The eight clusters in highest to lowest mean importance were:

i) Clearly communicate physical training program expectations and requirements (7 statements, mean importance = 3.70/5, mean feasibility = 3.75/5);
ii) Prepare for, monitor and manage physical training load (19 statements, mean importance = 3.69/5, mean feasibility = 3.43/5);
iii) Provide best practice injury identification, prevention and management (12 statements, mean importance = 3.69/5, mean feasibility = 3.43/5);
iv) Educate recruits, staff and other stakeholders involved in academy training delivery (14 statements, mean importance = 3.61/5, mean feasibility = 3.82/5);
v) Provide a supportive training environment that promotes health, wellbeing and injury reporting (15 statements, mean importance = 3.59/5, mean feasibility = 3.30/5);
vi) Have experienced staff deliver training and use appropriate equipment (8 statements, mean importance = 3.40/5, mean feasibility = 3.38/5);
vii) Deliver a comprehensive and holistic physical training program (16 statements, mean importance = 3.27/5, mean feasibility = 3.75/5),
viii) Have appropriate physical entry standards and requirements (5 statements, mean importance = 3.10/5, mean feasibility = 3.29/5).

### Statement Rating, Go-Zone and Pattern Matches

**Fig. 2:**
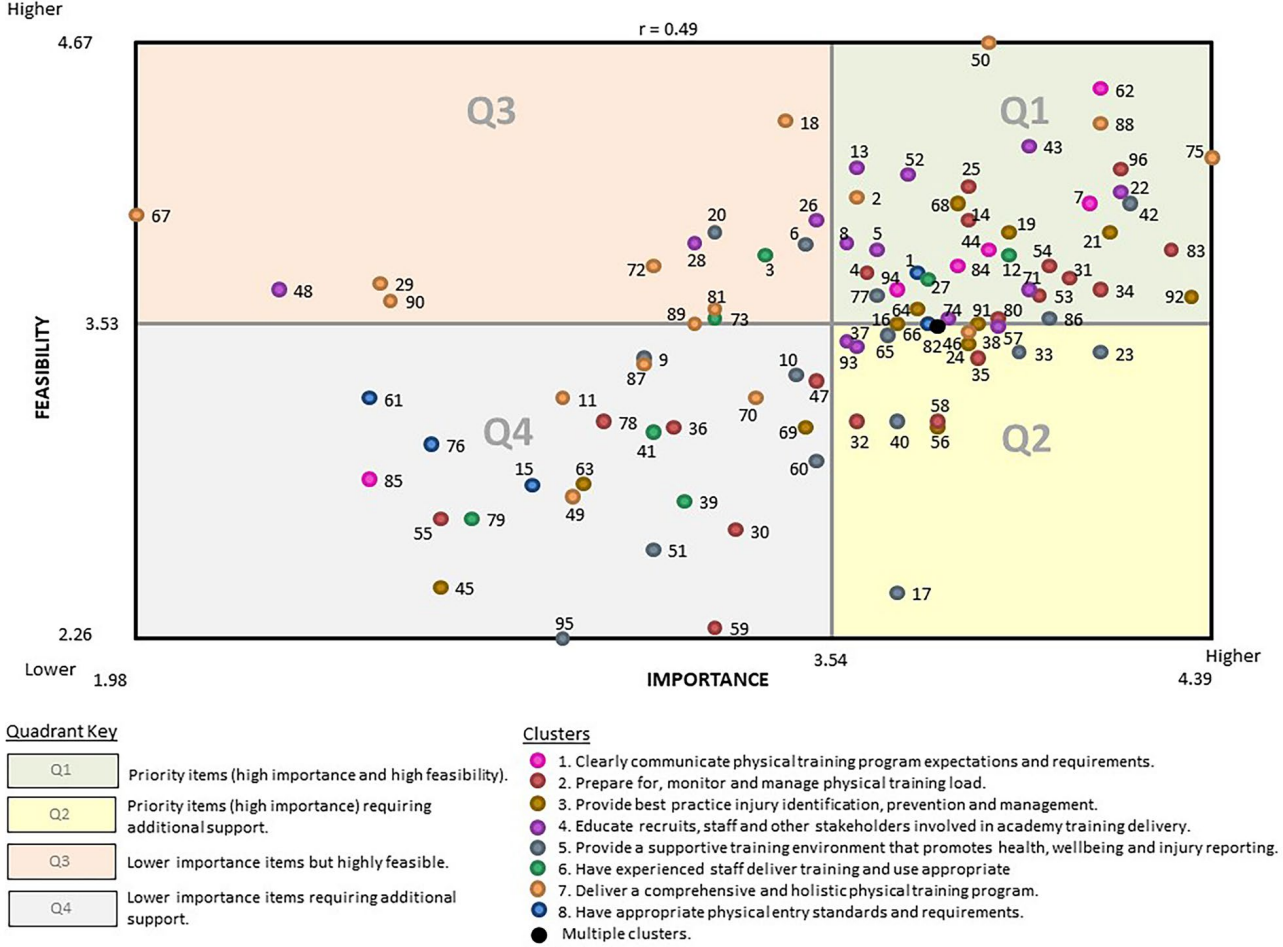
Go-Zone for individual statements for how to prevent injury and/or reduce the impact of injury on police force recruit training. Legend: This figure displays statements against feasibility and importance. Q1 shows 42 statements were above the grand mean for both importance and feasibility; Q2 shows 15 statements were above the grand mean for importance but below for feasibility; Q3 shows 14 statements were below the grand mean for importance but above for feasibility; and Q4 shows 25 statements were below the grand mean for both importance and feasibility

**Fig. 3:**
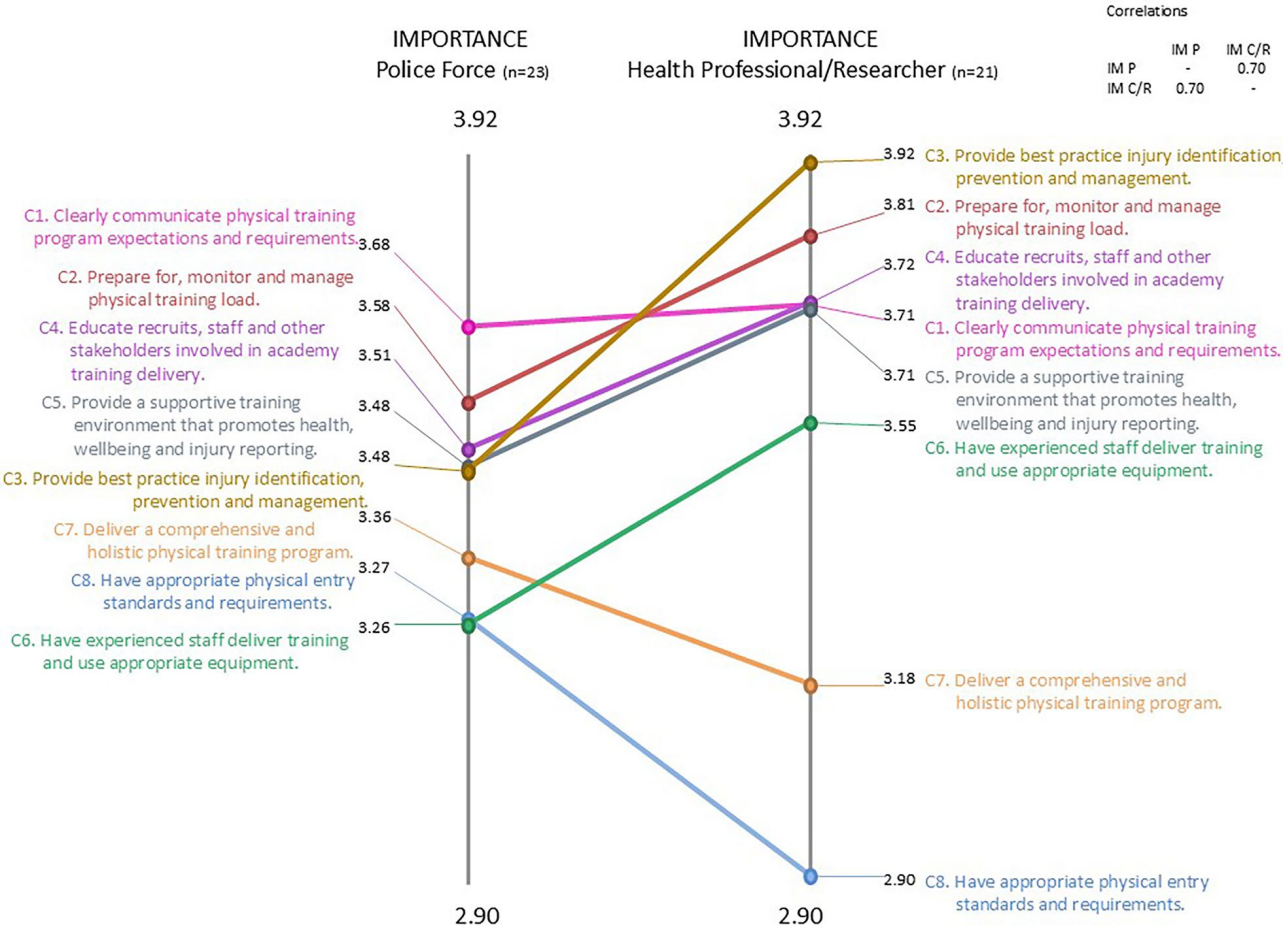
Differences in cluster importance between police force and health professionals/research experts. Legend: Pattern matching was used to visually compare mean cluster rating across participant subgroups (i.e. police force vs. clinician/researcher). When steep pattern match slopes suggested notable differences, Welch’s t-tests were applied to compare subgroup means within clusters. This test accounts for unequal variances and sample sizes, and cluster means were treated as interval-level data derived from item averages. N = number; IM = importance; P = police force recruits and staff; C/R = clinicians and research experts; C1 = Cluster One (and so forth)

**Fig. 4:**
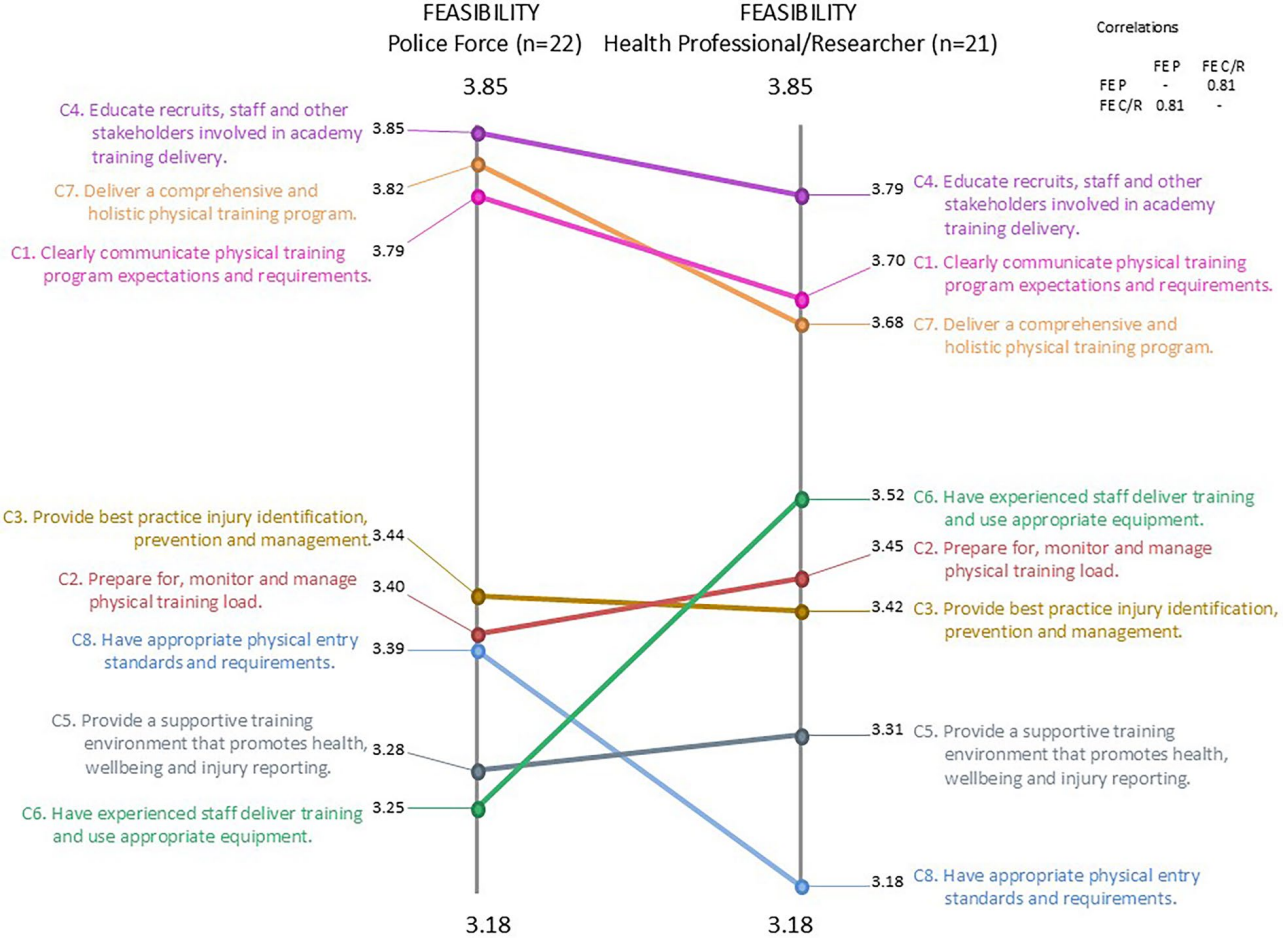
Differences in cluster feasibility between police force and health professionals/ research experts. Legend: Pattern matching was used to visually compare mean cluster rating across participant subgroups (i.e. police force vs. clinician/researcher). When steep pattern match slopes suggested notable differences, Welch’s t-tests were applied to compare subgroup means within clusters. This test accounts for unequal variances and sample sizes, and cluster means were treated as interval-level data derived from item averages. N = number; IM = importance; P = police force recruits and staff; C/R = clinicians and research experts; C1 = Cluster One (and so forth)

Forty-four participants rated the statements for importance, and 43 participants rated the statements for feasibility. A go-zone graph plotting the statements against importance (grand mean = 3.54) and feasibility (grand mean = 3.53) was generated (Fig. 2). Forty-two statements were above the grand means for importance and feasibility (Q1), fifteen statements were above the grand mean for importance but below for feasibility (Q2), fourteen statements were below the grand mean for importance but above for feasibility (Q3), and twenty-five statements were below the grand means for importance and feasibility (Q4). There were differences in the cluster mean importance (Fig. 3) and feasibility (Fig. 4) orders between the Police Force and health professionals/ research experts’ judgements. There was no difference detected between subgroups (Police Force versus health professionals/ research experts) for mean ‘feasibility’ of any of the clusters. There was one difference detected between subgroups (Police Force versus health professionals/ research experts) for the ‘importance’ of cluster three (Provide best practice injury identification, prevention and management), with health professionals/ research experts participants rating this as more important than Police Force participants (*p* < 0.05).

## Discussion

Using a concept mapping methodology (underpinned by a participatory research approach) [12], police force recruits or officers, police force staff, health professionals and research experts identified strategies they perceived could reduce the burden of injury in police force recruits. Participants generated 96 unique statements (42 were rated above the grand mean for importance and feasibility), which were agreed by the project steering committee, industry advisory group, and consumer advisory group [17] to be best represented by eight clusters (termed priority areas). These eight priority areas underwent a stepwise selection and naming process by the the project steering committee, industry advisory group, and consumer advisory group to ensure the priority areas reflected both prevention content and context expertise. Each of these priority areas included strategies that participants perceived to be above the mean rating for both importance and feasibility, to reduce the burden of injury in police force recruits.

### Preventing Musculoskeletal Injury in Recruits is Complex

The strategies identified fall within, and are influenced by factors within, a complex socioecological model [28]. Specifically, the strategies identified are relevant at individual (i.e. the recruit), interpersonal (e.g., physical performance or tactical staff delivering recruit training), organisational (e.g., police force training facility management or police force health and welfare staff), community (e.g., police force executive management from differing portfolios), and policy-maker (e.g., state and national government) levels. The importance of designing a prevention intervention to address different levels of the police force recruits’ socioecological system is underscored in both the most important priority area (Priority area 1: Clearly communicate physical training program expectations and requirements), and the most feasible priority area (Priority area 4: Educate recruits, staff and other stakeholders involved in academy training delivery). Further, to reduce the burden of injury, consideration should be given to primary, secondary, and/ or tertiary prevention strategies [29]. Whilst many of our clusters represent injury prevention priority areas in sport, there is little research for these priority areas in police recruit populations. Thus, our results reflect the complexities associated with preventing musculoskeletal injury caused by physical activity or sport, which are broadly acknowledged [30], with the eight priority areas identified in our study encompassing strategies from both primary and secondary prevention.

#### Primary Prevention

The bulk of important and feasible strategies provided by participants related to primary prevention. Given the physical demands of policing and recruit training, we anticipated participants would focus on physical prevention strategies. However, whilst some strategies were considered as more feasible and important [e.g., Strategy 2 (#2): strength exercises, #50 warming-up appropriately], many strategies involving physical elements that feature in existing musculoskeletal injury prevention programs [31] were considered less feasible and/ or important in the context of police recruit injury prevention (e.g., #72: agility training, #90: balance exercises, #29: stretching).

Preparing for, monitoring and managing physical training load was identified as the second most important priority area, which may be due to most injuries in police force recruits occurring early in police force recruit training programs [4]. The importance of avoiding large fluctuations in training load was identified (#34: scheduling, #31: load monitoring). Further, with the change in recruitment strategies over time, as the police force better reflects the community it is serving, it is also important to acknowledge existing training programs are historically catered to a population of predominantly young males, and may need to adapt as the focus of recruitment changes (e.g., an increase in the number of women recruits). Training load management is considered an essential element of successfully improving physical performance [32] and, whilst varying in context and magnitude, there is a recognised association between training load and overuse injury [33]. However, as the relationship between training load and injury is complex, manipulating it to reduce injury risk is subsequently challenging [34]. Further, quantifying training load is also a challenge, with recruit fatigue being influenced not only by the physical workload, but also contextual factors such as sleep or stress [35].

Priority areas 3, 4 and 5 propose important and feasible nutritional (#8: provide nutritional education) and psychosocial strategies, such as providing recruit training program staff with access to resources and expertise on mental health, psychology, and wellbeing to better support their recruits (#77). However, many of the psychosocial strategies related to mental fatigue and recovery were rated above average for importance, but not for feasibility (e.g., #35: providing adequate time for mental recovery, #32: providing flexibility within sessions based on mental fatigue, #56: monitoring recruit mental health, #93: education relating to holistic recovery strategies). Why these strategies were not rated as more feasible could be for many reasons, though we suspect one reason may be stigma related to mental health in police forces [36, 37]. This is problematic because certain psychological and sociocultural aspects of police force recruit training that parallell team sport have been associated with injury risk [38]. Additionally, organisational barriers, such as a rigid training structure without spare time to include recovery interventions to recruits, might have limited the perceived feasibility.

The priority area ‘have appropriate physical entry standards and requirements’ was rated as the least important and least feasible overall. This suggests that although addressing physical entry standards is a potential injury prevention strategy, there are more important and more feasible strategies that could be effective in reducing the burden of injury in police force recruits. We expect the lower feasibility is due to decisions related to entrance standards being implemented at organisational levels (and above). However, the lower importance was unexpected, given the published tactical recruit injury prevention literature (primarily military) has almost exclusively explored physical entry standards [8]. Whilst many factors (e.g., cardiorespiratory fitness or muscle strength endurance) have been identified as being associated with injury or recruit drop out, there have been no optimal cut-offs identified. We would propose that prior to suggesting amendments to physical entrance standards and requirements, if wanting to reduce drop out whilst maximising recruitment, the optimal cut-off point for physical entry standards and requirements should be determined.

#### Secondary Prevention

The majority of the most important and feasible strategies for secondary prevention related to systems that support recruits throughout injury recovery and encourage them to report injuries (#19). The strategies advocated included a range of experienced and well-qualified health professionals with experience in musculoskeletal injury management (#92), and ensuring shared decision-making between recruits, police force staff, and healthcare providers is in place (#21). These strategies work in similar environments. For example, the provision of healthcare providers during Air Force basic training has demonstrated a reduction in musculoskeletal injury related attrition [39], with a substantial economic benefit [40].

### Perceptions by Police Force Recruits and Staff Do Not Always Align with Healthcare Providers, and Research Experts

There were differences in how the police force and health professionals/research experts ordered the eight priority areas. Police force participants rated ‘provide best practice injury identification, prevention and management’ as less important than health professionals/research experts rated it. It is possible that the police force participants believed injury prevention and management strategies were already in place. Alternatively, health professionals/research experts may hold a conscious or unconscious bias regarding the importance of this priority area. Whilst no significant difference was detected when comparing sub-groups, when looking at the order of importance, police force participants rated ‘have experienced staff deliver training and use appropriate equipment’ as less important than health professionals/ research experts rated it. This may again be due to police force participants believing that this was already well established. It is also possible that the importance of equipment and physical training staff in preventing injury is underrecognised by police force staff and recruits.

When reviewing the order of feasibility for police force and health professionals/research experts, there were no statistically significant differences in rankings. However, there were differences in the order of importance. Police force participants rated ‘have appropriate physical entry standards and requirements’ higher than health professionals/research experts. Further, ‘have experienced staff deliver training and use appropriate equipment’ was rated higher in feasibility by health professionals/research experts than police force participants. Otherwise, there was little difference in the priority order between groups. One possible reason for the differences between groups is that one group (police force staff and recruits) has more accurate knowledge of the police force setting, which is why the inclusion of participants from the police force was so valuable.

### Future Directions

Our co-creation is guided by the Translating Research into Injury Prevention Practice (TRIPP) framework [41]. Previous research has established the epidemiology (step one) [4, 6], and aetiology (step two) [8]. Our mixed-method concept mapping approach has contributed to intervention development (step three). However, it is important to note that this is just one step in our intervention co-creation [14], with co-creation also guided by systematic reviews and further qualitative research [20].

We identified strategies to consider when developing an injury prevention intervention for police force recruits. However, concept mapping relies on participants’ *perceptions* of importance and feasibility, which may be influenced by individual roles and experiences rather than objective effectiveness. Therefore, our results must be considered alongside data relating to the effectiveness of the strategies proposed (as per previous clinical research), and a more detailed exploration of the contextual factors. This will enable to co-creation of a prevention intervention, in alignment with our established how-to-guide [14].

Systematic reviews and meta-analyses of the efficacy of all prevention interventions in recruit populations are needed. However, due to the dearth of literature in law enforcement recruit injury prevention, findings from comparable populations (e.g., military recruits or community sports) may be extrapolated to guide selection of effective strategies. To gain a deeper understanding of the contextual factors in a specific environment (e.g., a single police recruit jurisdiction) qualitative research involving police force staff and recruits is needed. This qualitative approach will explore what the prevention intervention will consist of by providing a better understanding of what prevention strategies are already in place, and how other strategies could be implemented. Subsequently driving the next stage of co-creation. Thus, our concept mapping approach, as well as further reviews of the literature and qualitative research should inform the development of a potentially effective prevention intervention, which can then be implemented and tested.

### Limitations

This study was co-designed and delivered via a collaboration between consumer, industry and research advisory groups, reducing the inherent biases from any one group [17, 20]. Further, through promoting diversity in participant inclusion [22], and application of the closeness continuum for healthcare providers and research experts, bias was limited. Police force staff and recruits were from a single police force jurisdiction, which from an implementation lens is a study strength, but may limit the generalisability of the results. The number of participants in our police recruit/officer group (n = 6) may appear small. However, > 10 of the police force staff group members were also police force officers so the number of participants who had lived experience of the recruit training program was > 15, representing the largest proportion of participants. All participants volunteered and this may introduce selection bias. Ratings were summarised using mean scores, as per established practice, but may not fully capture variability of responses. The results of Welch’s t-test should be interpreted cautiously. As such, we opted to analyse our sample into two groups (police force vs healthcare providers and research experts) as opposed to the four participant groups. Further, we discussed differences in the ‘order’ of the clusters between groups and did not just rely on the between-group statistical value.

## Conclusion

This concept mapping study was co-designed by consumer, industry and research advisory groups; ensuring lived experience and context expertise was included at all stages. Police force recruits or officers, police force staff, health professionals, and researchers identified eight broad injury prevention priority areas. These priority areas highlight the breadth of possible injury prevention approaches, and should directly inform the development of injury prevention interventions. The holistic and multifaceted nature of the statements suggest a broad mix of interventions targeting factors beyond individual physical fitness capacity, which are the responsibility of people at multiple levels of the police force ecosystem, can influence recruit injury burden.

## Data Availability

All non-identifiable data for this research project are available online (10.6084/m9.figshare.30486179).

## Appendix A: Demographic Data Variables

- Age (years)
- Sex recorded at birth (male/female/another term (please specify))
- Current gender (man or male/woman or female/non-binary/different term/prefer not to answer)
- Body mass index (BMI, calculated from self-reported height and weight)
- Ethnicity (self-reported)
- Country of primary residence (self-reported)
- Languages other than English spoken by the participant (self-reported)
- Whether the participant performs moderate to vigorous physical activity most days (yes/no)
- Highest level of education (less than high school degree/ high school graduate/ bachelor's degree/ master's degree/ doctoral degree/ other professional degree (e.g., Medical Doctor)
- Employment status (working full-time/ working part-time/ student/ homemaker or stay-at-home parent/ unemployed and looking for work/ retired/ other)
- Total household income (less than AUD $30,000/ between AUD $30,000—$49,999/ between AUD $50,000—$79,999/ between AUD $80,000—$99,999/ between AUD $100,000—$149,999/ between AUD $150,000—$199,999/ more than AUD $200,000/ prefer not to say)
- Nordic musculoskeletal screening (neck; shoulders; upper back; elbows; wrists/ hands; lower back; hips/ thighs; knees; ankles/ feet).
- Participants were then asked to select the group that best represented their study involvement (police force recruits and officers; police force staff; health professionals; research experts), with the inclusion criteria for each group provided.
- Participants self-reported their specific occupation (e.g., detective, medical doctor, physical trainer) and the number of months and years they have worked in their role.
- Police force recruits and officers also reported whether they have previously been employed in a tactical athlete role (e.g., in the military or other law enforcement agency).
- Research experts were asked to identify the injury prevention programs and contexts with which they have experience (e.g., knee injury prevention in soccer, lower back injury prevention in the military).

## Appendix C

See (Table

**Table 2:** Participant characteristics

	Police force recruit or officer (*n* = 6)			Police force staff (*n* = 19)			Health professional (*n* = 13)			Research expert (*n* = 10)		
	Median	Minimum	Maximum	Median	Minimum	Maximum	Median	Minimum	Maximum	Median	Minimum	Maximum
Age (Years)	34.0	24.0	44.0	44.0	29.0	60.0	37.0	28.0	53.0	48.0	32.0	65.0
Height (cm)	174.5	158.0	186.0	174.0	154.0	187.0	171.5	156.0	186.0	163.50	167.0	182.0
Weight (kg)	84.5	66.0	93.0	74.0	54.0	115.0	70.5	46.0	106.0	68.5	52.0	92.0
Pain Regions (n)	3.0	0.0	5.0	2.0	0.0	6.0	1.0	0.0	6.0	2.0	0.0	6.0
Work History (Years)	9.0	1.0	19.0	7.0	1.0	26.0	8.0	1.0	32.0	22.0	10.0	33.0

cm = centimeters, kg = kilograms, n = number

2).

## Appendix D

See (Table

**Table 3:** Strategies to prevent injury and/or reduce the impact of injury on police force recruit training (by cluster and in order of mean importance rating)

Cluster and statements Each statement is a continuation of the focus prompt: “To prevent injury and/or reduce the impact of injury on law enforcement recruit training, I think it’s important to…”		Bridging score^#^	Mean rating^*^ (± SD)		All statement go-zone
			Importance (n = 44)	Feasibility (n = 43)	
Cluster 1	Clearly communicate physical training program expectations and requirements (7 strategies)	0.54	3.70 (0.52)	3.75 (0.45)	–
62	Communicate physical fitness expectations within recruitment material	0.66	4.14	4.49	Q1
7	Raise awareness that minimum fitness standards are not high enough to prevent injury or meet the physical capacity required to complete the program, and set the bar high for recruits to rise to, rather than allowing recruits to only train to minimum standards	0.63	4.11	4.02	Q1
44	Ensure recruit training program staff have early knowledge of recruits' physical standards	0.65	3.89	3.84	Q1
84	Provide guidance to recruits on appropriate ways to monitor and increase training volume over time	0.37	3.82	3.77	Q1
46	Develop and provide pre-habilitation guidelines/recommendations/programs to recruits before starting the recruit training program	0.51	3.75	3.53	Q1
94	Undertake a generalised injury prevention program for everyone	0.52	3.68	3.67	Q1
85	Ask recruits what would be realistic for compliance regarding physical training, session duration and environment (e.g. gymnasium)	0.44	2.50	2.90	Q4
Cluster 2	Prepare for, monitor and manage physical training load (19 strategies)	0.22	3.69 (0.42)	3.43 (0.49)	–
83	Maintain standards of fitness across the entire duration of recruit training	0.19	4.30	3.84	Q1
96	Undertake physical tests that appropriately reflect workforce requirements (e.g. running in boots, carrying extra weight)	0.36	4.18	4.16	Q1
34	Ensure consistent physical training schedules to minimise large fluctuations in training load (e.g. four sessions one week, none the next)	0.14	4.14	3.67	Q1
31^**†**^	Monitor training load and volume within the program	0.16	4.07	3.72	Q1
54	Perform baseline screening for previous medical and injury history to inform recruit training	0.29	4.02	3.77	Q1
53^**†**^	Perform baseline strength, power and conditioning screening to inform training	0.10	4.00	3.65	Q1
80	Treat fitness requirements the same as critical skills training	0.22	3.91	3.56	Q1
35	Provide adequate time to recover physically and mentally in between drills that stress the same body area	0.32	3.86	3.40	Q2
14^**†**^	Create optimal baseline targets for strength/endurance	0.28	3.84	3.95	Q1
25	Undertake an annual review of training programs and testing methods	0.33	3.84	4.09	Q1
58	Review recruits' training and exercise history and adjust loading/requirements as appropriate	0.26	3.77	3.14	Q2
4	Gradually increase body armour load, instead of adding it all at once	0.30	3.61	3.74	Q1
32^**†**^	Provide flexibility within the sessions based on physical fatigue / injuries / mental fatigue	0.07	3.59	3.14	Q2
47	Provide recruits with sufficient time to train before starting the recruit training program	0.21	3.50	3.30	Q4
30	Use individual load management strategies that are holistic (based on both internal and external load measures)	0.11	3.32	2.70	Q4
59	Monitor and manage training load and volume of activities outside the training program (e.g. other sporting activities)	0.15	3.27	2.30	Q4
36	Avoid unofficial physical training sessions (e.g. from other areas of the recruit training program) that don't have a set structure or consider any load management	0.27	3.18	3.12	Q4
78	Commence formal tactical training later in the program	0.22	3.02	3.14	Q4
55	Test and monitor body composition (e.g. skeletal muscle mass and bone density) to inform training	0.30	2.66	2.74	Q4
Cluster 3	Provide best practice injury identification, prevention and management (12 strategies)	0.62	3.69 (0.20)	3.43 (0.44)	–
92	Use a range of experienced and well-qualified health professionals with experience in musculoskeletal injury management	0.52	4.34	3.64	Q1
21	Support injury return by joint decision-making and shared responsibility between health care professionals, the recruit and police instructors	0.61	4.16	3.91	Q1
19	Have a system in place to support recruits throughout the injury recovery process (e.g. communication pathways, opportunity to modify training content)	0.58	3.93	3.90	Q1
91	Have appropriate pathways and management for recruits at high-risk of ongoing injury burden or when recovery is not progressing as planned	0.58	3.86	3.53	Q1
24	Ensure physical trainers are supervised by and have access to appropriately qualified/accredited support staff (e.g. physiotherapists, exercise physiologists, occupational physicians)	0.56	3.84	3.45	Q2
68	Employ clear goal setting post injury and monitor progress	0.57	3.82	4.02	Q1
56	Monitor and manage the mental health of recruits throughout recruit training	0.77	3.77	3.12	Q2
64	Employ a full-time physiotherapist based at the recruit training program	0.56	3.73	3.60	Q1
16	Provide a harmonious, supportive, and inclusive social environment to ensure recruits feel valued and supported within the training program	0.90	3.68	3.53	Q1
69	Identify psychological (e.g. anxiety, depression) and social (e.g. work satisfaction) risk factors	0.64	3.48	3.12	Q4
63	Have an on site triage area to identify non-injuries	0.57	2.98	2.88	Q4
45	Use podiatrists as necessary, to ensure appropriate footwear recommendations	0.6	2.66	2.47	Q4
Cluster 4	Educate recruits, staff and other stakeholders involved in academy training delivery (14 strategies)	0.22	3.61 (0.43)	3.82 (0.26)	–
22	Educate recruits, physical trainers and law enforcement staff on the importance of early injury recognition and intervention	0.13	4.18	4.07	Q1
43	Provide education on injury reporting (what, who, when)	0.15	3.98	4.26	Q1
71	Educate all stakeholders involved about injury prevention programs, including officers, recruits and other stakeholders of relevance	0.35	3.98	3.67	Q1
57	Understand the patterns, cause and nature of the injury/ies	0.53	3.91	3.52	Q2
74	Complete mandatory education on how to safely address and reduce the impact of injuries before starting the recruit training program	0.28	3.80	3.56	Q1
52	Educate recruits on recovery, including sleep hygiene	0.08	3.70	4.14	Q1
5	Educate recruits about common soft-tissue injuries, and how/why they happen	0.12	3.64	3.84	Q1
13	Provide education about the expected response to physical training (e.g. muscle soreness vs. muscle injury)	0.08	3.59	4.17	Q1
93	Educate about and facilitate access to holistic recovery strategies (psychological, social, mental, and physical)	0.20	3.59	3.44	Q2
8	Include nutrition education (e.g. daily nutrition needs, meal preparation, injury recovery, reduce inflammation, etc.)	0.36	3.57	3.86	Q1
37	Complete mandatory education on how to optimise their performance and technique before starting the recruit training program	0.19	3.57	3.47	Q2
26	Provide dietary and nutrition advice (e.g. for weight management, adaptation to training, to avoid sarcopenia and maintain/build muscle mass.)	0.41	3.50	3.95	Q3
28	Educate about the importance of managing (e.g. posture adjustment) and minimising load carriage (e.g. removing body armour as often as practical)	0.13	3.23	3.86	Q3
48	Provide education on seated and computer ergonomics	0.08	2.30	3.67	Q3
Cluster 5	Provide a supportive training environment that promotes health, wellbeing and injury reporting (15 strategies)	0.63	3.59 (0.37)	3.30 (0.51)	–
42	Ensure adequate training of the physical training instructors	0.55	4.20	4.02	Q1
23	Build and maintain a culture that enables recruits to report physical complaints early (e.g. not seeing injuries as being "weak" or "soft")	0.80	4.14	3.42	Q2
86	Provide qualified and experienced strength and conditioning coaches to supervise, program, and deliver physical training sessions	0.55	4.02	3.56	Q1
33	Ensure good relationships between recruits, physical trainers, exercise physiologists, physiotherapists and occupational physicians engaged within the recruit training program	0.66	3.95	3.42	Q2
17	Ensure that sworn police force officers maintain physical fitness to show recruits that it is crucial to being a police officer	0.55	3.68	2.44	Q2
40	Monitor and address factors, such as fatigue, distress, wellbeing, are addressed, so that recruits can be their best self and fully attentive	0.54	3.68	3.14	Q2
65^**†**^	Support recovery post-strenuous activities (e.g. nutrition, rest, sleep, ice baths, massage, etc.)	0.49	3.66	3.49	Q2
77	Provide recruit training program staff with access to resources and expertise on mental health, psychology, and wellbeing to better support their recruits	0.73	3.64	3.65	Q1
60	Undertake individualised assessment to provide appropriate advice about weight distribution of vest, body armour, handcuffs, baton, firearm, taser, and other accessories	0.55	3.50	2.98	Q4
6^**†**^	Support adequate hydration (fluid intake) and electrolyte levels	0.50	3.48	3.86	Q3
10	Encourage rest and recovery when 'niggles' or small, unassuming injuries are present	0.53	3.45	3.33	Q4
20	Identify environmental conditions that may impact injury risk or burden (e.g. wet surface)	0.58	3.27	3.91	Q3
51	Screen for possible disordered eating (e.g. low energy availability) and knowledge of food intake	0.60	3.14	2.62	Q4
9	Have injured recruits describe their experiences, on how they wish they had better managed things within their control	0.94	3.11	3.40	Q4
95	Provide them with an individualised nutrition plan to support them throughout training	0.80	2.93	2.26	Q4
Cluster 6	Have experienced staff deliver training and use appropriate equipment (8 strategies)	0.88	3.40 (0.37)	3.38 (0.41)	–
12	Complete all pre-medicals and baseline testing by recruit training program staff	0.63	3.93	3.81	Q1
82	Provide high quality personal protective equipment for tactical, physical, and combat training	0.99	3.77	3.52	Q2
27	Identify specific equipment that may impact injury risk or burden (e.g. obstacle course, weight of vest or belt)	0.91	3.75	3.71	Q1
3	Avoid using physical training as a punishment	0.98	3.39	3.81	Q3
73	Allow recruits to wear recommended footwear rather than mandated specific footwear for training	1.00	3.27	3.56	Q3
39	Provide good quality, well-fitting footwear as part of recruit uniform issue	0.99	3.20	2.81	Q4
41	Have recommended external personal trainers who understand physical entry requirements and can work with prospective recruits	0.84	3.14	3.10	Q4
79^**†**^	Use wearable devices to track recruits' movements and provide real-time feedback on posture and form	0.73	2.73	2.74	Q4
Cluster 7	Deliver a comprehensive and holistic physical training program (16 strategies)	0.16	3.27 (0.61)	3.75 (0.47)	–
75	Use training which reflects required functional activities of law enforcement officers	0.07	4.39	4.21	Q1
88	Design training programs that build functional strength and fitness	0.14	4.14	4.35	Q1
50	Warm-up appropriately (including dynamic movements) for all training content	0.01	3.89	4.67	Q1
38	Provide a strength and conditioning program before entering the recruit training program for those identified as not up to physical standard	0.54	3.84	3.50	Q2
2	Focus on strength exercises	0.01	3.59	4.05	Q1
18	Include proper cool down routines	0.13	3.43	4.36	Q3
70	Provide recovery weeks based on body adaptations to training	0.32	3.36	3.23	Q4
81	Include more variety of cardiorespiratory exercises (e.g. more low-impact movements such as cycling, rowing machine, skiing, etc.) rather than just running	0.00	3.27	3.60	Q3
89	Build a solid base of core strength early, before higher loads of the upper and lower body are considered	0.05	3.23	3.53	Q3
72	focus on agility training exercises	0.02	3.14	3.77	Q3
87	Consider posture and fundamental movement skills as part of routine training to encourage healthy movement at all times	0.18	3.11	3.37	Q4
49	Get successful applicants to contractually agree to continue on a set training plan, until entering the recruit training program	0.59	2.95	2.83	Q4
11	Introduce foot and ankle muscle strengthening in the physical training program early	0.00	2.93	3.23	Q4
90	Focus on balance exercises	0.02	2.55	3.63	Q3
29	Use stretching techniques/yoga	0.20	2.52	3.70	Q3
67	Use foam rolling	0.25	1.98	3.98	Q3
Cluster 8	Have appropriate physical entry standards and requirements (5 strategies)	0.47	3.10 (0.54)	3.29 (0.32)	–
66	Increase the physical entrance test standard (e.g. higher beep test level)	0.51	3.75	3.53	Q1
1^**†**^	Complete recruits' final physical assessment closer to when they enter the recruit training program	0.43	3.73	3.74	Q1
15	Adjust physical entrance standards to account for male/female differences	0.50	2.86	2.88	Q4
76	Adjust physical entrance standards to account for age differences	0.45	2.64	3.05	Q4
61	Review the maximum recruitment age	0.47	2.50	3.23	Q4
All statements		–	3.54	3.53	–

All statement go-zone: Q1 = above all-statement mean on importance and feasibility; Q2 = above all-statement mean on importance, below all-statement mean on feasibility; Q3 = below all-statement mean on importance, above all-statement mean on feasibility; Q4 = below all statement mean on importance and feasibility

^#^Values range between 0.00 and 1.00. Values closer to 0 indicate anchoring statements closely related to others in the cluster. Values closer to 1 indicate bridging statements more connected to statements in other clusters in the map

^*^1 = low; 5 = high

^†^Reassigned from an adjacent cluster

3).

## Appendix E

See (Table

**Table 4:** Amendment to cluster names following consumer and industry partner review

Cluster number	Original cluster names from the steering committee	Revised cluster names following consumer review	Revised cluster names following industry partner review
1	Clearly communicate program expectations and requirements	Keep	Clearly communicate physical training program expectations and requirements
2	Prepare for, monitor and manage training load	Prepare for, monitor and manage physical training load	Keep revision
3	Provide best practice injury identification and management	Keep	Provide best practice injury identification, prevention and management
4	Educate recruits and staff involved in training delivery	Educate recruits and staff involved in academy training	Educate recruits, staff and other stakeholders involved in academy training delivery
5	Provide an environment that supports health and wellbeing	Merge: Provide an environment that supports health, wellbeing and injury reporting	Provide a supportive training environment that promotes health, wellbeing and injury reporting
	Culture and reporting		
6	Have expert personnel and appropriate equipment	Have expert physical trainers and appropriate equipment	Have experienced staff deliver training and use appropriate equipment
7	Deliver a comprehensive physical training program	Keep	Deliver a comprehensive and holistic physical training program
8	Have appropriate physical entry standards and requirements	Keep	Keep

4).
